# Cross cultural and global uses of a digital mental health app: results of focus groups with clinicians, patients and family members in India and the United States

**DOI:** 10.1017/gmh.2021.28

**Published:** 2021-08-24

**Authors:** Elena Rodriguez-Villa, Abhijit R. Rozatkar, Mohit Kumar, Vikram Patel, Ameya Bondre, Shalini S. Naik, Siddharth Dutt, Urvakhsh M. Mehta, Srilakshmi Nagendra, Deepak Tugnawat, Ritu Shrivastava, Harikeerthan Raghuram, Azaz Khan, John A. Naslund, Snehil Gupta, Anant Bhan, Jagadisha Thirthall, Prabhat K. Chand, Tanvi Lakhtakia, Matcheri Keshavan, John Torous

**Affiliations:** 1 Division of Digital Psychiatry, Department of Psychiatry, Beth Israel Deaconess Medical Center, Harvard Medical School, Boston, USA; 2 All India Institute of Medical Sciences Bhopal, Bhopal, Madhya Pradesh, India462020; 3 Department of Global Health and Social Medicine, Harvard Medical School, Boston, USA; 4 Sangath, Bhopal, Madhya Pradesh, India462016; 5 National Institute of Mental Health and NeuroSciences, Bangalore, India560029

**Keywords:** global health, digital health, mental health, smartphone apps, mhealth, informatics

## Abstract

**Background:**

Despite significant advancements in healthcare technology, digital health solutions – especially those for serious mental illnesses – continue to fall short of their potential across both clinical practice and efficacy. The utility and impact of medicine, including digital medicine, hinges on relationships, trust, and engagement, particularly in the field of mental health. This paper details results from Phase 1 of a two-part study that seeks to engage people with schizophrenia, their family members, and clinicians in co-designing a digital mental health platform for use across different cultures and contexts in the United States and India.

**Methods:**

Each site interviewed a mix of clinicians, patients, and their family members in focus groups (*n* = 20) of two to six participants. Open-ended questions and discussions inquired about their own smartphone use and, after a demonstration of the mindLAMP platform, specific feedback on the app's utility, design, and functionality.

**Results:**

Our results based on thematic analysis indicate three common themes: increased use and interest in technology during coronavirus disease 2019 (COVID-19), concerns over how data are used and shared, and a desire for concurrent human interaction to support app engagement.

**Conclusion:**

People with schizophrenia, their family members, and clinicians are open to integrating technology into treatment to better understand their condition and help inform treatment. However, app engagement is dependent on technology that is complementary – not substitutive – of therapeutic care from a clinician.

## Background

Global demand for access to mental health care is growing today despite access collapsing (Kuehn, 2020). Rising suicide and depression rates reflect an exigent, unmet need for innovative treatment options (Torous and Wykes, 2020). Despite significant advancements in healthcare technology, digital health solutions for mental health continue to fall short of their potential across both clinical practice and in terms of clinical efficacy (Lagan, 2020*a*, 2020*b*). The utility and impact of medicine, including digital medicine, hinges on relationships and trust, particularly in the field of mental health (Torous and Roberts, 2017). For individuals living with serious mental illness, such as schizophrenia spectrum disorders, establishing trust when using digital mental health interventions is especially important given this vulnerable population often faces significant stigma (van Zelst, 2009). This stigma can have a detrimental impact on the quality of care they receive and may vary by culture and region (Krendl and Pescosolido, 2020; Yin *et al*., 2020). Conversely, many mental health care providers report that digital health tools are seldom created with their needs in mind, and that it is not feasible to integrate many apps into routine clinical care settings (Bucci *et al*., 2019; Lattie *et al*., 2020). Creating and assessing digital tools that can realize the full potential of digital health and overcome these barriers is especially critical now as the field accelerates use of these technologies in light of coronavirus disease 2019 (COVID-19) (Hasson-Ohayon and Lysaker, 2020; Maguire and Looi, 2020).

As access to and use of smartphones and mobile internet continues to increase in many lower resource settings globally, including in underserved regions of higher income countries such as the United States, as well as many regions in low-income and middle-income countries (LMICs) such as India, there may be new opportunities to extend the reach of digital mental health interventions. In India, for example, mobile Internet penetration continues to increase rapidly reaching close to 450 million people by 2023 (Kokane *et al*., 2019). Yet, alarmingly, there are significant gaps between those living with mental illnesses and those who have access to adequate care in India (Naslund *et al*., 2019) where in excess of 90% of individuals living with mental disorders do not have access to care in many regions in the country (Maulik *et al*., 2017). Prior studies have demonstrated that digital technology can effectively support task sharing mental health services in various low-resource settings, through the use of digital tools for supporting frontline health workers with diagnosis, guiding clinical decision making, and facilitating supervision (Merchant *et al*., 2020). In India, there have been recent successful efforts to leverage technology such as smartphone apps, for supporting community health workers in primary care settings, and raising awareness about mental health within rural villages (Muke *et al*., 2020). However, as highlighted in a recent review, there is limited evidence for using smartphones to support care for individuals with severe mental disorders, such as schizophrenia spectrum disorders (Powell *et al*., 2019). These unmet needs are compounded by the fact that a 2020 review of the commercial app stores (Apple and Google) found only two schizophrenia specific apps developed with links that have been published in publicly accessible scientific literature (Lagan *et al*., 2020*a*).

To address this gap and to inform the implementation of digital solutions that are both relevant to the target patient groups and grounded in sound clinical evidence, it is necessary to engage multiple stakeholder groups early in the design and development process. Recently, studies have recorded and analyzed attitudes towards integrating technology into mental health care (Berry *et al*., 2017). Papers around the co-design of mental health apps are also emerging around schizophrenia (Allan *et al*., 2019; Berry *et al*., 2020; Realpe *et al*., 2020) but these all focus on tools that are not publicly available or either focus on insights from either care providers or care recipients – but not both. No studies that we are aware of sought to translate apps across cultures and health systems as distinct as India and the United States. Smartphone Health Assessment for Relapse Prevention (SHARP) is an international, digital mental health study funded by the Wellcome Trust with the goal of increasing access to evidence based and personalized mental health education and treatment (Rodriguez-Villa *et al*., 2021). The overarching goal of this two-phase, observational research study is to measure the efficacy and impact of an open-source platform and smartphone application, mindLAMP (Torous *et al*., 2019) in preventing relapse among individuals with schizophrenia spectrum disorders.

This paper details the methods and results of Phase 1 of SHARP to aggregate insights from mental health clinicians, their family members, and people with schizophrenia in co-designing a digital mental health platform for use across different cultures and contexts in the United States and India. Specifically, in this paper, we report a multi-site qualitative study exploring the needs of both mental health care providers, people with schizophrenia, and their families, and highlight opportunities for utilizing technology towards creating trust, sustaining engagement, and ensuring efficacy in future clinical use cases. In conducting this work and publicly sharing the mindLAMP app, we aim for results presented here to be broadly generalizable and serve as a foundation for others seeking to expand, improve, and iterate upon our efforts.

## Objective

The purpose of Phase 1 is to engage clinicians, and people living with schizophrenia spectrum disorders and their family members, from three study sites distinct in culture and setting – the Beth Israel Deaconess Medical Center (BIDMC) in Boston, USA, the All India Institute of Medical Sciences (AIIMS) in Bhopal, India, and the National Institute of Mental Health and Neurosciences (NIMHANS), Bengaluru, India – in developing new features and co-designing the mindLAMP app. To understand the diverse needs of all stakeholders and to find harmony around use of the same app in three distinct settings, we sought to identify the features and functions most appealing as well as learn what changes to the content or layout are necessary. The feedback and themes collected from focus groups at all three sites will inform both front end user interface and content, as well as technical modifications to mindLAMP in an iterative manner. Collecting data from each site will also help ensure that the application is deployed in a manner that is culturally meaningful in distinct settings, offering a unique viewpoint of how one app can be customized to meet diverse local needs (Torous and Vaidyam, 2020). In Phase 2 of this project, the mindLAMP app will be used in a clinical study to assess its effectiveness in predicting and preventing relapse in schizophrenia at the same three sites.

## Methods

### Procedures

We employed thematic analysis based on grounded-theory approach (Turner, 1981) seeking to capture the actions, interactions, and processes related to use of the mindLAMP app in relapse prevention. While many qualitative methods exist, grounded theory has been suggested for use in guiding app development for underrepresented mental health consumers (Leung *et al*., 2016) and has been employed for technology development for schizophrenia as well (Lindberg, 2017; Gumley *et al*., 2018). Participants in Phase 1 of SHARP included clinicians, family members, and people with schizophrenia to represent a theoretically chosen sample to best inform a complete theory. For the purpose of this study, clinicians were defined as clinically trained mental health care professionals. Starting with open categories based on prior qualitative research and feedback from ongoing use with the mindLAMP app, as well as an in-person discussion and use of the app by all three teams in February 2020, data from focus groups and its analysis were collected and conducted simultaneously and iteratively (Torous *et al*., 2019; Nebeker *et al*., 2020; Saldaña, 2021).

**Fig. 1.**
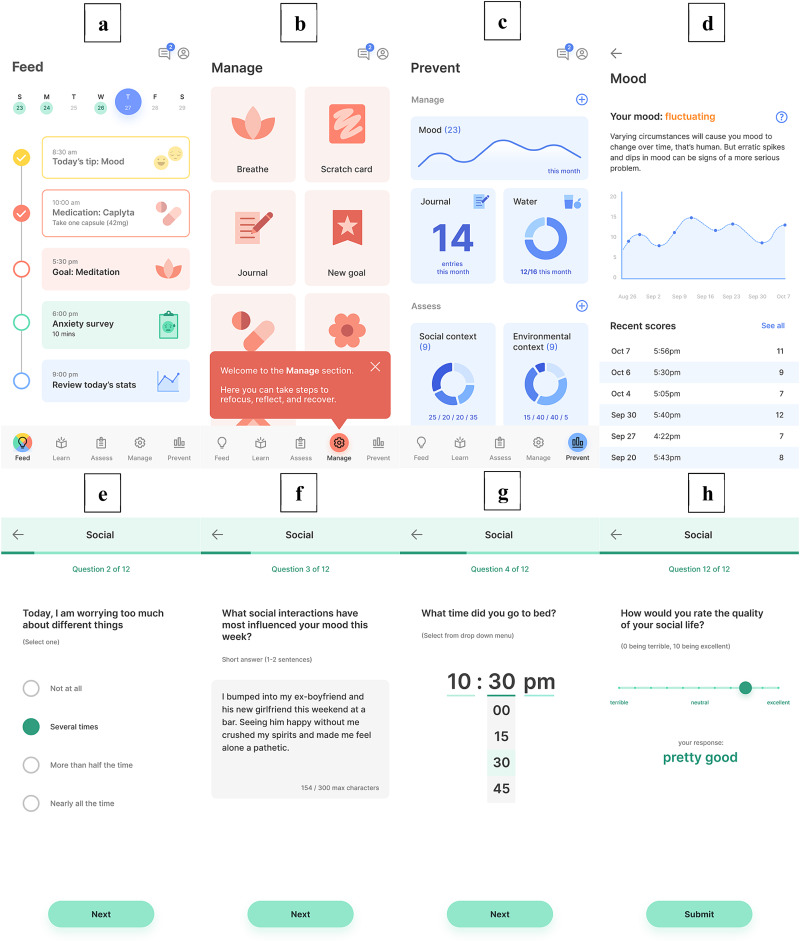
A visual overview of select portions of the mindLAMP app; (a) the Feed displays upcoming tasks and reminders; (b) the app is divided into Learn, Assess, Manage, and Prevent sections, each containing a different set of activities; (c) the Prevent section displays a simplified visual overview of data; (d) when a data tile is tapped, a detailed view of each data point is presented, along with a simplified textual interpretation of the chart; (e–h) the Assess section presents surveys of varying length, such as the Social survey in this example, with modifiable questions and answer choices.

We selected sample sizes larger than similar grounded theory work seeking insight into designing technology systems for use in schizophrenia (Lindberg, 2017; Gumley *et al*., 2018) with the goal of ensuring saturation would occur. Sites continued focus groups until saturation occurred as determined by the study authors, acknowledging there is simple definition of saturation (Braun and Clarke, 2021). Prior to beginning the focus groups, all teams in India and Boston agreed on key exploratory questions focused on understanding how stakeholders experience and would interact with mindLAMP. Iterative data collection, documentation of ideas and emerging trends, and analysis through open coding of categories was used to develop a set of central themes. See Appendix 1 for a script of the interview questions and Fig. 1 for examples of sample screens shared in focus groups and interviews. Participants in focus groups were recruited through online flyers and word of mouth recruitment among clinicians. The app was translated into Hindi for focus groups taking place in India, although the English version was also used.

All sites received ethics approval from respective Institutional Review Boards. To protect the health of participants and accommodate social distancing during the COVID-19 pandemic, the study site at the BIDMC conducted all focus groups and interviews over Starleaf – a HIPAA compliant videoconferencing platform, enabling virtual meetings. At the study sites in Bhopal and Bengaluru, focus groups were conducted both in-person and through videoconferencing. Sites in India audiotaped focus group discussion for obtaining raw data for transcription.

All focus groups and interviews across sites followed the same format. After an overview of the meeting's agenda and verbal consent given by participants for virtual focus groups and written informed consent for in-person focus groups, staff members of the research team asked participants to describe their own smartphone use. Follow-up questions and discussion were guided by interests and experiences unique to participants but addressed activities and attitudes related to general topics summarized in Table 1.

**Table 1.** Topics of discussion in focus groups and interviews prompted participants to consider how and why they use their smartphone

Discussion framework
Health – mindfulness tools, physical activity tracking, self-reflection and journaling, mood tracking, sleep tracking
App engagement – adopting new apps, when apps are used, why apps are not used, notifications
Work/life balance – communication with clinicians/people with schizophrenia, appointment scheduling, email
Telehealth – transition to teletherapy, platform usability, privacy, remote work

After open discussion, research staff demonstrated relevant components of the mindLAMP platform. Participants were shown the dashboard, a component of the mindLAMP platform that stores and displays patient data. All were shown the mindLAMP smartphone application that collects patient data through surveys, games, and actigraphy sensors. During the session, participants were allowed to download and use the app in order to better assess its user interface and interactive features, or the app was projected onto a screen for the moderator's ease of explaining navigation and app features. To facilitate access, we created a computer-based version of the app to allow anyone to interact with and navigate mindLAMP. Research staff encouraged participants to give specific feedback on the usefulness of features, the look and feel of the design, and their understanding of the platform's functionality throughout the demonstration. Participants were also given the opportunity to ask questions and offer comments at the conclusion of the demonstration. Research teams emphasized that adaptations to mindLAMP were ongoing and invited participants to continue to share insights or apposite experiences with digital health after the meeting.

### Participants

#### BIDMC

Six focus groups for mental health care providers were conducted by the BIDMC study site. Focus groups ranged in size from two to six participants. Each participant was asked to complete a survey about their technology use before the meeting. A total of 20 mental health care providers participated in focus groups with BIDMC, ranging in ages 26 to 63 with a mean age of 41. The mental health care providers included in this study represent a range of training, expertise, and responsibilities. A detailed breakdown of clinical roles of the mental health care providers that participated in the focus groups at each site is outlined in Table 2.

**Table 2.** Focus group participants at BIDMC, AIIMS, and NIMHANS represented a range of roles, backgrounds, and responsibilities of mental health care providers and number of family members and people with schizophrenia

Provider title	BIDMC total	AIIMS total	NIMHANS total
Nurse practitioner	1	3	2
Social worker	5	3	3
Psychiatry resident	5	2	0
Psychiatrist	9	2	10
Psychologist	0	2	5
People with schizophrenia and family members	25	25	25

**Fig. 2.**
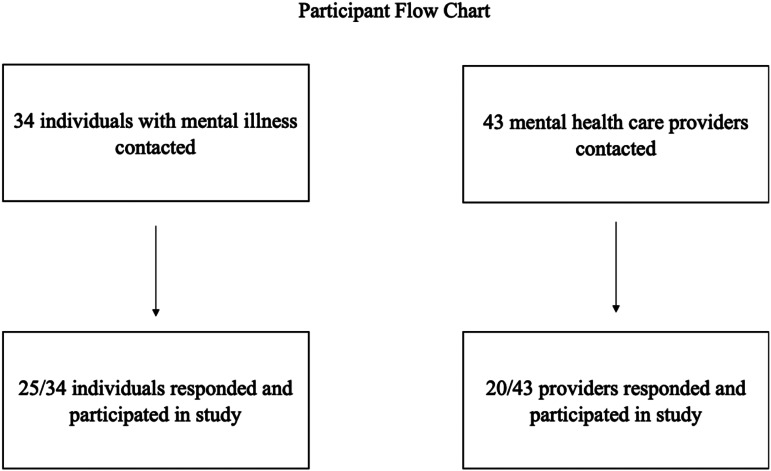
A total of 25 individuals with mental illness and 20 mental health care providers participated in interviews and focus groups at the BIDMC. All participants completed their interview or focus group. They were not contacted for follow-up.

Research staff also interviewed a combined total of 25 individuals with mental illness and their family members at BIDMC. These participants were drawn from those in care with BIDMC and thus illness was not assessed as an inclusion criterion. Interviews followed the same format as focus groups and were conducted one-on-one to protect confidentiality. people with schizophrenia's ages ranged from 18 to 65 with a mean age of 32 years. Interviews with these participants focused on their personal experience utilizing the mindLAMP smartphone app and salient opportunities for improvement (Fig. 2).

#### The AIIMS, Bhopal

Three focus groups for mental health care providers were conducted by research team at AIIMS, Bhopal. A total of 13 mental health care providers participated in these focus groups with ages ranging from 23 to 73 years old with a mean age of 35 years.

Research staff also held focus groups for a total of 25 individuals with mental illness and their family members. These participants were drawn from those in care with AIIMS and thus illness was not assessed as an inclusion criterion. people with schizophrenia's ages ranged from 23 to 42 years old with a mean age of 33 years, and family members’ ages ranged from 23 to 72 years old with a mean age of 50 years.

#### National Institute of Mental Health and Neuro-Sciences, Bengaluru

Eleven focus groups for mental health care providers were conducted by research staff at NIMHANS. A total of 20 mental health care providers participated in these focus groups with ages ranging from 23 to 72 years old with a mean age of 41 years.

Research staff also held focus groups for a total of 25 individuals with mental illness and their family members. These participants were drawn from those in care with NIMHANS and thus illness was not assessed as an inclusion criterion. people with schizophrenia's ages ranged from 25 to 46 years old with a mean age of 35 years, and family members’ ages ranged from 26 to 66 years old with a mean age of 41 years.

Focus groups and interviews across study sites were documented via audio-recordings, handwritten notes, and typed notes which were used for content and thematic analysis guided by grounded theory outlined in Methods. After focus groups concluded, each site coded emerging and extant themes. All coding of transcripts was conducted by at least three authors at each site and consisted of the same steps: breaking down each transcript into discrete categories or themes; removing duplicate, redundant, and non-relevant responses; mapping remaining themes; selecting representative quotes that could be used to represent that point; and sharing the current results across calls with all three teams in Boston, Bhopal, and Bengaluru. The focus groups and interviews took place over 5 months in October 2020–February 2021 with weekly calls between the three research site teams.

## Results

### Similarities in results among research sites

Several themes emerged across all research sites, including: increased use and interest in technology use during COVID-19, need for safe and trusted data use and sharing, and supporting app engagement through human interactions. We present significant findings below that reflect and connect these distinct themes. These findings helped inform adaptations to the mindLAMP app (Table 3).

1. COVID-19 led to an uptake of virtual therapy, presenting new challenges and opportunities for providers and people with schizophrenia, using technology.

**Table 3.** Adaptations to mindLAMP were informed by feedback and insights that related to recurring findings themes of (1) Technology use during COVID-19, (2) Data use and sharing, and (3) App engagement

Specific adaptation to mindLAMP	Common themes
In-app messaging	Technology use during COVID-19
Mood monitoring	Technology use during COVID-19
Screen time monitoring	Technology use during COVID-19
Access hierarchy	Data use and sharing
Detailed manual for new users	Data use and sharing
Passive data visualization	Data use and sharing
Increased survey customization	App engagement
Custom images	App engagement
Available languages	App engagement

Focus groups with providers were held during the coronavirus outbreak. In light of social distancing mandates in Massachusetts, all providers in focus groups at the BIDMC had transitioned to virtual meetings with people with schizophrenia and colleagues. New tools such as Zoom, Google Meet, and StarLeaf were common platforms providers at BIDMC had adopted that enabled them to videoconference. Most providers had not used these platforms to meet with people with schizophrenia prior to the pandemic. The adoption of videoconferencing was described by many as ‘forced’, but most providers felt comfortable using the new tools after one or two meetings as well as now open to further new tools like smart-phone based or digital apps.

In discussions, providers agreed that videoconferencing allowed them to connect with people with schizophrenia and these virtual sessions made it possible for people with schizophrenia to continue treatment. However, many providers brought up new challenges in now offering online care. The most frequent concern was privacy. At home with family members and roommates in close proximity, clinicians worried that people with schizophrenia may not be alone and thus less comfortable or willing to speak candidly. Moreover, some providers felt uncomfortable ‘entering’ a patient's space and conversely, felt uncomfortable with a patient entering theirs. Nearly all providers felt that videoconferencing made it more difficult to interpret body language and pick up on nonverbal cues. Providers who work with children, in particular, explained that virtual visits make it more difficult to hold a patient's attention and that it is challenging to eliminate distractions and control external stimuli. Providers noted that smartphone data could offer a new information source that would enable them to compensate for what could be lost via video visits. However, providers noted that they would want to understand regulations and liability governing access and use of this data – citing they are not able to respond to constant data streams that could be possible with mindLAMP data.

Many people with schizophrenia brought up videoconferencing when asked how they use their smartphone. Similar to providers, most people with schizophrenia had not met with their therapist over a digital platform prior to COVID-19. Attitudes towards virtual visits differed. One person with schizophrenia said that facetiming with his therapist was *‘better than nothing but not nearly as good as in person’* where he is *‘able to read them* [*the therapist*]*’* and ‘*not distracted by* [*his own*] *image on the screen’.* Another person with schizophrenia, however, said that videoconferencing was *‘almost better’* because he was less focused on pleasing his therapist and was less distracted by their presence.

1. Access to data may offer providers and people with schizophrenia new insight into illness and treatment, but too much data elicit discomfort.

Unique to the mindLAMP platform is its capacity to collect, store, and display a range of patient data. The mindLAMP smartphone application collects a combination of **active** and **passive** data from people with schizophrenia. Active data include responses to mood and symptoms surveys, while passive data accumulate metrics related to physical activity and phone use from device sensors. In this sense, active data are subjective and determined by the patient while passive data are objective and collected without any direct input from the patient. A visual overview of the mindLAMP platform can be found in Fig. 1. Both providers and people with schizophrenia described access to some combination of active and passive data as ‘*valuable*’.

Among providers, several were interested in eeing passive data from people with schizophrenia, specifically metrics on sleep patterns and step count. *‘It*'s *an indirect measure of some* (people with schizophrenia's) activity. Which patient may *not tell but you will get some inference from the indirect sources*’, commented a provider, highlighting how passive data presents a new source for information on people with schizophrenia's activities and an alternative to self-reported activity and symptoms. Providers also expressed that collecting passive data longitudinally might make it easier to track the efficacy of brief interventions. However, when shown examples of aggregated passive data, most providers wanted less information available to them. Comments such as *‘I feel overwhelmed with data to begin with’* and ‘*this is too much data’* were echoed across focus groups. Although mindLAMP does not track people with schizophrenia's GPS location in real time but instead tracks their movement overtime, some providers were uneasy about accessing this information. ‘*I don't want to know exactly where they go*’, a provider reiterated.

People with schizophrenia expressed more excitement viewing their own active data. Specifically, pat people with schizophrenia wanted to *‘see what the pattern is’* in how their mood or symptoms fluctuated. Most people with schizophrenia explained that seeing how their survey answers changed day to day would help with *‘creating awareness’* and *‘engaging with the things that were happening in life’.* Tracking mood or symptoms appealed to most people with schizophrenia Some people with schizophrenia added, however, that seeing fluctuations was only as helpful as follow up with action items. *‘I would want there to be something I could easily take action on’,* said one person with schizophrenia.

People with schizophrenia did not express discomfort with their data being collected, noting they felt that there were strong protections in place to ensure their privacy. A handful of people with schizophrenia, however, described how looking at data may impact how they answer future surveys. One person with schizophrenia said that if they could see their answers aggregated as data points, they would be motivated to answer surveys with more regularity. *‘It would incentivize me to be more accurate’,* a person with schizophrenia explained. Conversely, another person with schizophrenia worried that they would answer surveys dishonestly in an attempt to improve a trend in a behavior or mood they were able to track over time. Some providers echoed this concern and worried that self-report surveys might be completed inaccurately. Some people with schizophrenia also commented that they did not want to be reminded or shown data that reflected a depressed or anxious state.

Although one person with schizophrenia remarked that passive data collection was *‘low key freaky’,* people with schizophrenia for the most part did not express concerns about their privacy. *‘I know that my phone knows everything about me. Why not get it to help me?’* remarked one person with schizophrenia.

1. Relevance and integrated experience increase engagement.

When asked how they used their smartphone, providers and people with schizophrenia listed similar features and applications. Providers and people with schizophrenia described the most engagement with native applications – apps available on their smartphone that do not require download. Among these were email, texting, calendar, voice calls, and alarm settings. More people with schizophrenia than providers cited social media, games, banking, and exercise tracking tools as frequently used apps. Many people with schizophrenia mentioned apps they had downloaded or used to support mindfulness and healthy habits. However, the extent to which people with schizophrenia engaged with and utilized mindfulness applications was limited. ‘*I don't have a good habit of doing it regularly, but when I do its pretty useful’,* a person with schizophrenia described about the regularity of their use of a commercial application that offers meditation modules. ‘*Most of the time, I'll download it and stop using after 2 days’,* said another person with schizophrenia about applications they download from the app store.

Many people with schizophrenia explained that they learned about mindfulness or mental health-related apps through word of mouth. Oftentimes, they downloaded an app following a recommendation from their provider. This aligned with focus group discussion among providers, many of whom had recommended applications to people with schizophrenia. Providers and people with schizophrenia alike admitted that user reviews and privacy policies had little to no impact on whether they recommended or downloaded an app – citing lack of time as reasons for not examining apps in more detail. While some providers had recommended apps focused on clinical outcomes such as Dialectical Behavioral Therapy diary card or Cognitive Behavioral Therapy applications, most providers had suggested mindfulness applications to people with schizophrenia as a means to minimize anxiety and help refocus energy as well as apps for lifestyle modification such as weight loss apps. Most providers did not formally follow up with people with schizophrenia about their experiences with or continued use of a recommended application.

For many people with schizophrenia, monotony and impersonal content deterred regular use of an application. *‘Each time I go back, I'd have to feel like I'm seeing something new’,* a person with schizophrenia elaborated. People with schizophrenia described intuitive navigation, accessible language, an aesthetically pleasing design, and a sense of reward as contributing factors towards high engagement. Most people with schizophrenia agreed that notifications increased the likelihood that they opened an application and that ‘*being reminded to use it consistently*’ spurred habitual use.

Providers and people with schizophrenia also emphasized the importance of training and education to optimize app use. *“I think for using the app* [*mindLAMP*], *there should be some initial, let us say, starting lessons or starting guides… ‘how to use this app’”.* They remarked that generally an app is only useful if a user understands its features and how to utilize them, and so that the more comfortable a provider or patient is navigating mindLAMP, the more likely they will be to use it.

Notably, all people with schizophrenia have expressed interest and excitement towards using a smartphone application in conjunction with therapy. Most people with schizophrenia said that they would be willing – and that they would like to – share data collected on mindLAMP with their therapist directly through the app and or during in-person therapy. By filling out surveys and tracking specific moods or behaviors, they would be able to record patterns and recall feelings they might otherwise neglect or forget in between clinical visits. *‘I have a week in between therapy sessions. I find it really easy to lose the momentum and not do the work outside of therapy. I feel like it would help me carry that momentum from one therapy session to the next’,* a person with schizophrenia explained. People with schizophrenia echoed this sentiment and furthered that the surveys would be a useful way to update providers and contextualize conversations.

### Differences in results among research sites

Attitudes towards technology and feedback specific to mindLAMP were far more similar in sites in the United States and India than they were different, suggesting that there is potential for digital tools to support healthcare globally. There were, however, comments and topics of discussion that differentiated findings in the United States and India.

1. Participants in India discussed government policy

In discussions related to data privacy and the overall safety of mindLAMP, government recommendations or guidelines were not brought up by a single participant – provider or person with schizophrenia – at BIDMC. However, in India, some providers in focus groups were employed by the government (public sector) and noted that this reporting structure impacted how they are permitted to interact with people with schizophrenia. *‘Because we are all from the government setting, so we don't have that freedom and that copyright of seeing the patient the way we want. Prescribing the patient, the way we want. Right? So, there are definitely some restrictions as per government policy’.* They noted, however, that these ‘restrictions’, were not the same for providers in private practice.

Relatedly, people with schizophrenia in India wanted to understand how they ensure the *‘legality’* and ‘*authenticity’* of the doctor they would hypothetically connect to via mindLAMP. This concern was not raised by people with schizophrenia in the focus groups at BIDMC.

1. Diverse uses for mindLAMP were discussed in India.

At sites in Bhopal and Bengaluru, participants suggested more novel and varied uses for mindLAMP than in Boston. These included using mindLAMP as a means to: access healthcare services including legal and ambulatory services, consult a doctor, and provide educational information to doctors. Participants noted how mindLAMP could be used as both a self-help tool, as well as a tool to monitor positive and negative symptoms unique to schizophrenia or any given illness – mental or physical.

1. Smartphones are not ubiquitous in rural areas in India.

mindLAMP is a tool only available to smartphone users. Thus, studies or providers that utilize it are only successful if people with schizophrenia have regular access to a smartphone. People with schizophrenia and families might share a smartphone, and or they have limited connectivity and available data. Providers in India outlined this important clause, mentioning that in ‘*certain studies which we have conducted, we have found out that unavailability of the smartphone and not able – not having the knowledge to use the smartphone – was a problem’.* Providers cautioned that ‘*Definitely, in rural areas it will be difficult*’, to ensure mindLAMP use and engagement. To mitigate this issue, providers suggested conducting local induction sessions to teach basic smartphone skills, demonstrate mindLAMP features, and make technical support available to offer help.

## Discussion

### Implications of findings

Our results are in line with a current focus in the field of digital mental health around understanding success of smartphone apps and implementation. The results here suggest broad interest in using apps in schizophrenia with common themes including adapting apps into the healthcare delivery in light of COVID-19, ensuring app data are used to benefit care, and increasing engagement through human support which parallels factors that facilitate implementation (Connolly *et al*., 2020) including external factors related to healthcare systems, internal factors related to care delivery, and a focus on the users, respectively. Previous qualitative studies have yielded similar results, highlighting openness among providers but also pervasive concern related to workflow and restrictions and accessing digital tools (Theme 1) (Lattie *et al*., 2020). While providers in this study were apprehensive about opening new lines of direct communication with people with schizophrenia (Theme 2), they reported more interest in relation to technologies in response to COVID-19 and social distancing mandates. All parties felt human support with mindLAMP is critical to engagement (Theme 3). Our results focus on people and connections as most critical for success, findings in line with recent implementation science research exploring why local efforts related to behavioral nudging often fail at scale (Bird *et al*., 2021). The implications of these findings reinforce the need to invest not only in mental health technology but also the systems and people necessary to support it.

All participants agreed that COVID-19 had made them more interested in using apps for care because (1) barriers to trying new technology are fewer and (2) new app data could supplement telehealth visits to make the visits richer and bring outside information into the online session. Participants noted that mindLAMP would be most useful if it could integrate with an electronic medical record and offer a direct link between the app and clinical encounter and access to medication information. Among all participants, there were fewer concerns related to privacy and safety than we anticipated based on our own prior research with mindLAMP. This finding may be indicative of a new landscape for digital health that the world is adapting to (Theme 1). Restricting in-person meetings has necessitated providers to learn and leverage more digital tools to carry out routine clinical responsibilities. In this new landscape, there may be more opportunity to introduce and integrate technology into care. While the impact of COVID-19 certainly influenced our results in terms of increasing interest in mobile health, it is likely this interest is neither merely reactive nor transient. Across the world interest in mobile health has increased (Inkster, 2021) and COVID-19 will continue to influence mental health care delivery even after the world population is vaccinated (Torous and Wykes, 2020).

Based on results from Theme 1, we believe that specific and tailored education and instruction for using mindLAMP will optimize its use. Clinicians and people with schizophrenia will be more inclined to use mindLAMP to supplement care if they realize its benefits and can navigate the platform with confidence. To this end, in future iterations of the mindLAMP platform we will pilot test a few strategies, such as providing users with an informational video and a detailed manual that includes step-by-step instructions and frequently asked questions for clinicians and researchers as well as people with schizophrenia and participants, respectively. We will also create PDF summaries of data which can be generated on demand and determine the feasibility of uploading these details into medical records as an immediate solution to integration. Further work to allow mindLAMP to integrate with medical records using the SMART and FHIR standards will continue.

Participants were excited by use of the new data and care resources afforded by mindLAMP, but also raised concerns about use of the data to improve outcomes. Participants reported limited experiences with digital mental health applications. Their understanding of data informed treatments that could be piloted with mindLAMP was limited and conceptual. All parties noted are not accustomed to accessing and viewing the range and extent of smartphone data mindLAMP can collect, such as step count or sleep data in routine care settings. While the fact that mindLAMP can share results in real time was appreciated, there remained confusion from providers and people with schizophrenia alike on how to act on that data.

Informed by Theme 2, we will create guidelines that help researchers interpret app data and explain and share it with people with schizophrenia. We will also update the app so that it is possible for clinicians to create custom views so that data visible to them and shared with people with schizophrenia can be as limited or extensive as the clinician and person agree is appropriate or helpful. For example, if a provider is only interested in viewing how many steps their patient is taking daily, they can customize the platform to display these metrics alone or in conjunction with other selected datapoints. Adaptations to mindLAMP will also include building a journal feature so people with schizophrenia can track their mood and record their thoughts to contextualize data for themselves and their clinician.

The efficacy of mindLAMP hinges on engagement and our results suggest people – not technology – will continue to drive that engagement (Theme 3). Building on trust, providers are poised to recommend technology to their people with schizophrenia and support continued use. Moreover, they can help people with schizophrenia utilize their own data to identify patterns and reflect on behavioral tendencies. If people with schizophrenia utilize mindLAMP in tandem with ongoing treatment from a provider, they may be encouraged to complete more surveys and provide clinicians further context and timely information. This could enable a reciprocal relationship between people with schizophrenia and clinicians, where information shared by people with schizophrenia through use of mindLAMP could prompt targeted feedback and recommendations from providers. In such a scenario, use of technology holds potential to strengthen the therapeutic relationship and enable remote support for continuity of care.

### mindLAMP in India

Certain results were unique to India. Focus groups in India suggested wider uses of mindLAMP beyond relapse prevention in schizophrenia, thus, highlighting the broad potential of mobile health tools like smartphone apps in other LMICs. Additional regulatory concerns appear specific to the rapidly changing telehealth policies in India – with the first national framework introduced in June 2020 and new to nearly all at the times of the focus groups. Issues of access to smartphones in more rural communities reflect an important consideration that may limit universal access to mindLAMP. Global trends in access to smartphones and wireless coverage however, suggest such disparities will continue to shrink in coming years.

An advantage of technology, specifically smartphone apps, is the ability to carefully tailor content to different languages and cultural contexts through systematic engagement of local stakeholders and partnership with the local health system, as demonstrated in prior work conducted by our team in rural India (Naslund *et al*., 2019). mindLAMP has been translated to Hindi and Kannada – with help from research collaborators in Bhopal and Bengaluru to ensure cultural relevance – so that the app can be used by providers and people with schizophrenia in a language they are comfortable using in the context of healthcare. Towards cultural relevance, active user engagement, and efficacy, study sites will continue to collect insight to inform further adaptations to mindLAMP during Phase 2.

While there is increasing interest in using mobile technology to advance mental health in LMICs (Ridley *et al*., 2020), there are few other studies to directly compare this project to. A 2-week feasibility study of a passive data smartphone app in Nepal reveled similar findings around the need to prioritize privacy and ensure support for engagement (Maharjan *et al*., 2021). Early work in Ghana highlights interest and potential for smartphones to advance mental health in this region with access and interest both high (Kola *et al*., 2021). While our results around use of the mindLAMP app in India and the United States cannot be generalized to other countries, the broad themes uncovered are likely relevant.

### Study limitations

Participants in focus groups – clinicians as well as people with schizophrenia and family members – elected to partake in the study. Our results may reflect that these participants were interested in technology and or already using digital tools and resources to supplement care. We did not re-verify diagnosis of people with schizophrenia as they were each in treatment with the respective site the study was conducted at. In this study, many people with schizophrenia and their family members participated in joint focus group discussion. This may have impacted their comfort level sharing phone habits, expressing concerns over privacy, and raising other issues that conversation may have elicited. Finally, nursing practitioners and psychologists were underrepresented in our sample although critical users who we envision will have active roles in supporting and engaging with technologies like mindLAMP.

To protect the privacy of focus group participants and adhere to the protocol of our ethical approval, recorded comments and feedback from clinicians, people with schizophrenia, and family members were not linked to individuals. Thus, our results and thematic analysis did not account for age or gender – demographics that may inform or impact attitudes towards technology.

This research was conducted during the COVID-19 pandemic, October 2020–February 2021, and initial plans for in-person research had to be adapted to online formats. While this did not decrease the rigor of the study, we did not conduct the study utilizing any checklists like the consolidated criteria for reporting qualitative research (Tong *et al*., 2007). We were also unable to gather data on how many participants were screened to partake at the India sites.

## Appendix 1: Similar questions were used to guide discussion and collect feedback from clinicians and patients. Discussion prompts for clinicians are outlined below.

### Focus group discussion prompts – clinicians

**I. General Phone Use**

1. What do you use your phone to do?
2. What are some apps you have on your phone that you like or don't like? Why?
3. How often do you use those apps?
  1. Which apps in particular do you use the most?
  2. When do you use them?
  3. How much time do you spend using them?
4. Have you always used them? Do you remember why you started using them to begin with?
5. How did you learn to use these apps?
6. How long did it take you to become familiar with them?
7. How do you decide whether or not to continue using an app?

**II. Apps for Health/Care**

*For your patients*:

1. Have you recommended any apps to your patients before?
  1. Which apps have you recommended?
  2. What made you recommend them (high ratings, recommended by a doctor/friend, looked cool etc)
  3. Did you get feedback from your patients around whether or not they used them? Did they like them?
  4. Do you feel like any app has contributed to an improvement in your patient's mood, functional outcomes, cognition etc?
    1. What was the app?
    2. What did you notice?
2. What areas do you believe an app could be most helpful in for your patients? Are there any areas or topics that would be most helpful to particular patient population you work with?
  1. Sleep
  2. Medication adherence
  3. Stress / Anxiety
  4. Connecting with peers and support
  5. Distractions (games)
  6. Mood tracking (journaling)

***For yourself as their clinician***:

1. What do you see as the most time-consuming or inconvenient parts of your day-to-day work?
2. How do you use technology in your work?
3. How do you communicate with patients outside of face-to-face interaction?
  1. How did this change during the COVID19 crisis?
  2. What technology did you begin using, if any?
    1. What did you like about it?
    2. What would you have changed about it?
4. Do you use any apps to support your practice?
  1. If yes, which app?
    - How and why did you choose to use it?
    - How often do you use it?
    - What in particular does it help you with?
    - What are its most useful functions or features?
  2. If no, why not?
5. What information, if any, about your patients daily activity are you curious about?
  1. What would be useful to know about your patients that you don't already?
6. Do you communicate with other people that support your patients (this can mean primary care physicians, family, pharmacists)?
  1. If yes,
    1. Who/what is their role?
    2. Through what medium do you communicate with them?
    3. How often do you communicate with them?
7. How do you log or keep track of patient treatment plans and / or sessions?
  1. Do you share this information with your patients?

**III. mindLAMP**

*Begin with 10-minute demonstration that includes: answering surveys, showing customizations, games (Jewels and the Box Game), the Scratch Card. After demonstration, give participants 5 minutes to practice answering surveys on their own and browsing the app.*

1. What did you think of the mindLAMP app? What are your first impressions?
2. What do you like or dislike about mindLAMP app?
3. Do you like the way it looks?
4. Is it easy to use?

***For your patients*:**

1. Describe how you think a patient might benefit from LAMP
  1. Do you see it as more useful for a particular illness or diagnosis?
  2. Do you see it as more useful for a particular age demographic?
2. What are some of the things you would change to the app?
  1. What would features would you take away?
  2. What features related to physical or mental illness would you add?
3. What are your reservations around the app?
4. Would you feel comfortable suggesting a patient use the app? Why or why not?
  1. If not, what would you change to make yourself feel more comfortable recommending LAMP?

***For yourself as their clinician:***

1. What features or functions feel more relevant to you? Why?
2. Would you feel comfortable use the app? Why or why not?
